# What is your diagnosis?

**DOI:** 10.4274/jtgga.galenos.2020.2019.0202

**Published:** 2020-12-04

**Authors:** Kavita Khoiwal, Payal Kumari, Om Kumari, Jaya Chaturvedi, Prashant Durgapal

**Affiliations:** 1 Department of Obstetrics and Gynecology, All India Institute of Medical Sciences, Rishikesh, India; 2 Department of Pathology, All India Institute of Medical Sciences, Rishikesh, India

An unmarried girl aged 17 years presented to our outpatient department with abdominal distension, dull aching abdominal pain, and amenorrhoea of three months duration. She attained menarche at 15 years of age and her previous menstrual cycles were regular, with average flow. General physical examination was within normal limit. Abdominal examination revealed a firm, non-tender, mobile abdomino-pelvic mass corresponding to 22 weeks of uterus size. Ultrasound (USG) showed a large solid cystic right adnexal mass with internal septations. In view of suspicion of ovarian malignancy, tumor markers were ordered. The serum values of CA-125 (28.5 U/mL), CA-19.9 (24.6 U/mL), carcinoembryonic antigen (0.67 ng/mL), alpha fetoprotein (1.5 IU/mL), and human chorionic gonadotropin (1.2 mIU/mL) were within normal limits and lactate dehydrogenase (LDH) (403 U/L) was raised. CECT abdomen and pelvis (Figure 1) suggested a well-defined, solid, multi-cystic abdomino-pelvic mass lesion (20.7x14.6x14 cm) arising from the right adnexa with enhanced septations and hyper dense component, which was thought to be probable ovarian adenocarcinoma. The right ovary was not evidently separate. The uterus was normal in size and the left ovary was not clearly visible. These findings led to a high clinical suspicion of ovarian malignancy and a plan of conservative staging laparotomy with right salpingo-ovariotomy was made in conjunction with the oncology department.

## Answer

**Figure 2:**
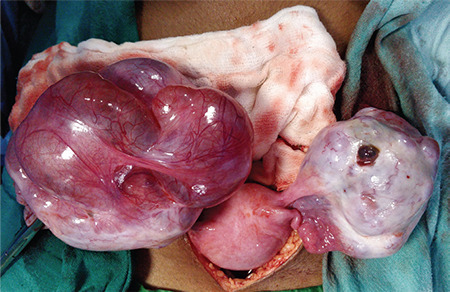
Intra-operative image suggestive of bilateral ovarian masses, an 8x6 cm solid cystic left ovarian mass and a 10x8 cm multi cystic right ovarian mass

**Figure 3:**
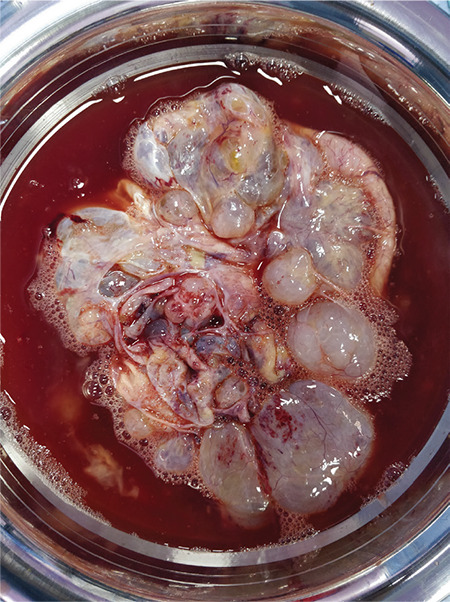
Cut specimen of left ovarian mass showing both solid and cystic areas containing clear fluid and a solid white-colored area

**Figure 4:**
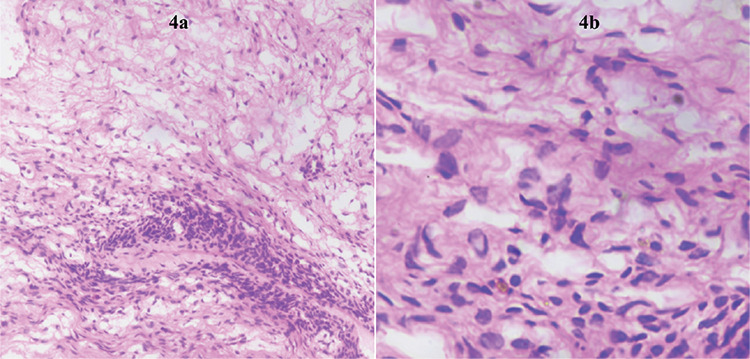
Hematoxylin and eosin (x10 and x40) stained section showing ovarian parenchyma with markedly loose and oedematous stroma, luteinisation of follicular cells, areas of hemorrhage and no atypia, suggestive of massive ovarian edema

Laparotomy was performed and intraoperative findings were suggestive of bilateral ovarian masses, an 8x6 cm solid, cystic, left ovarian mass and a 10x8 cm multi-cystic, right ovarian mass (Figure 2). The left-sided mass appeared to be malignant with no salvageable ovarian tissue. Left salpingo-ovariotomy and right sided cyst drainage, followed by excision of cyst wall was performed. The cut section of the left ovarian mass showed both solid and cystic areas containing clear fluid, and a solid, white colored area (Figure 3). The right ovary had multiple, clear, fluid-filled cystic areas. Intraoperative frozen section was suggestive of serous cystadenoma. Her postoperative course was uneventful. Final histopathological examination (HPE) of the left ovary and right ovarian cyst wall revealed ovarian parenchyma with markedly loose and oedematous stroma, luteinisation of follicular cells, areas of hemorrhage and no atypia, suggestive of massive ovarian edema (MOE) (Figure 4).

MOE is defined by the World Health Organization as an accumulation of edema fluid in the stroma, separating normal follicular structures (1). It is a rare entity, first reported in 1969 by Kalstone et al. (2).

Most of the cases have occurred among reproductive age group women but have also been reported in a 6-month-old girl and in a post-menopausal woman (3,4). Almost 85% of these cases are unilateral and bilateral MOE is rarer (5). Patients usually present with abdominal pain in conjunction with palpable adnexal mass (6). Hormonal symptoms, such as menstrual irregularity, precocious puberty, infertility and virilization may be concurrent, due to stromal hyperplasia (5).

The exact pathogenesis of MOE is still unknown but may be because of partial or complete ovarian torsion, secondary to PCOS, fibrothecoma, or metastatic carcinoma, all of which have been reported in the literature (2,3,4,7,8,9). When there is no underlying ovarian pathology, it is known as primary MOE. In the present case there was no evident underlying cause, and thus this is a case of primary MOE.

USG findings are inconclusive in most of the cases. Magnetic resonance imaging (MRI) has been found to be successful in diagnosing MOE, which shows an enlarged ovary with follicles around the ovary (10). In our case, the USG was inconclusive and CECT scan could not detect MOE. MRI was not requested as we did not suspect MOE. This is the rationale for publication of this case, as high clinical suspicion and awareness of the disease is crucial for optimal management.

Tumour markers are usually normal although raised LDH and CA-125 have been found in caes of ovarian edema with Meig’s syndrome and fibrothecomas (8,9).

Although there may be a preoperative and intra-operative suspicion of MOE, the final diagnosis is made only on HPE. MOE usually mimics ovarian malignancy, which results in over-treatment with salpingo-ovariotomy. The mainstay of treatment is wedge resection of ovary (5). A high index of suspicion is crucial for correct diagnosis and to conserve fertility. Risk of recurrence and long term implications of MOE are yet to be studied.

MOE is a rare ovarian disorder mimicking ovarian malignancy. Most cases present in young girls and are over treated. Awareness of the disease and a high index of suspicion is the key to successful outcome.

## Figures and Tables

**Figure 1:**
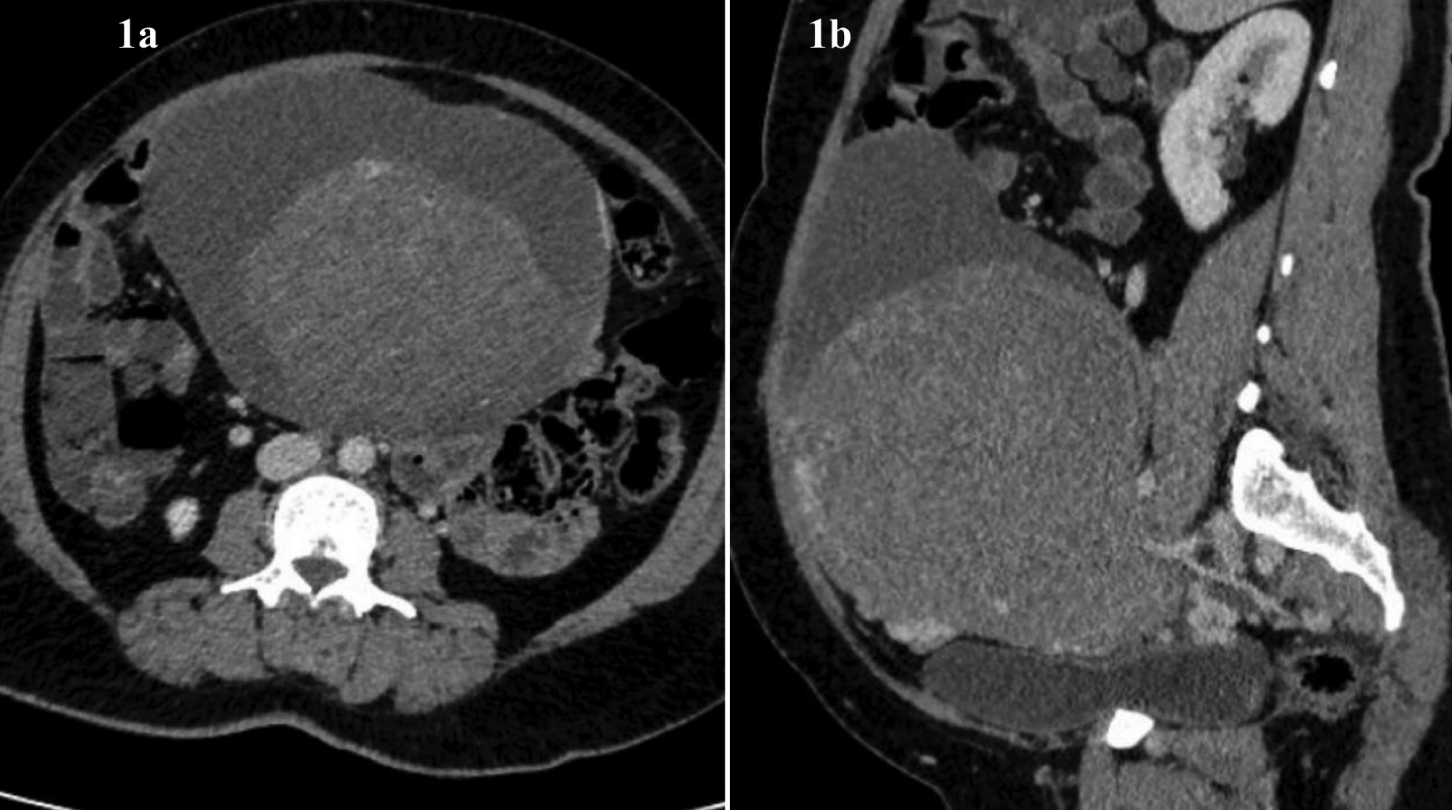
CECT abdomen & pelvis showing a well defined solid cystic lesion of size 20.7x14.6x14 cm arising from right adnexa with solid component measuring 18x14x14 cm (a) Transverse section, (b) Sagittal section
